# Enhancing the Longevity and Functionality of Ti-Ag Dry Electrodes for Remote Biomedical Applications: A Comprehensive Study

**DOI:** 10.3390/s23198321

**Published:** 2023-10-08

**Authors:** Daniel Carvalho, Sandra Marques, Giorgia Siqueira, Armando Ferreira, João Santos, Dulce Geraldo, Cidália R. Castro, Ana V. Machado, Filipe Vaz, Cláudia Lopes

**Affiliations:** 1 Physics Centre of Minho and Porto Universities (CF-UM-UP), University of Minho, 4710-057 Braga, Portugalclaudialopes@fisica.uminho.pt (C.L.); 2 LaPMET—Laboratory of Physics for Materials and Emergent Technologies, University of Minho, 4710-057 Braga, Portugal; 3 Chemistry Centre, University of Minho, 4710-057 Braga, Portugal; 4 Institute for Polymers and Composites, University of Minho, 4800-058 Guimarães, Portugal; 5 Polymer Engineering Department, Institute for Polymers and Composites, University of Minho, 4800-058 Guimarães, Portugal

**Keywords:** biopotential, dry electrodes, lifespan, Ti-Ag thin films, degradation, voltammetry

## Abstract

This study aims to evaluate the lifespan of Ti-Ag dry electrodes prepared using flexible polytetrafluoroethylene (PTFE) substrates. Following previous studies, the electrodes were designed to be integrated into wearables for remote electromyography (EMG) monitoring and electrical stimulation (FES) therapy. Four types of Ti-Ag electrodes were prepared by DC magnetron sputtering, using a pure-Ti target doped with a growing number of Ag pellets. After extensive characterization of their chemical composition and (micro)structural evolution, the Ti-Ag electrodes were immersed in an artificial sweat solution (standard ISO-3160-2) at 37 °C with constant stirring. Results revealed that all the Ti-Ag electrodes maintained their integrity and functionality for 24 h. Although there was a notable increase in electrical resistivity beyond this timeframe, the acquisition and transmission of (bio)signals remained viable for electrodes with Ag/Ti ratios below 0.23. However, electrodes with higher Ag content (Ag/Ti = 0.31) became insulators after 7 days of immersion due to excessive Ag release into the sweat solution. This study concludes that higher Ag/Ti atomic ratios result in heightened corrosion processes on the electrode’s surface, consequently diminishing their lifespan despite the advantages of incorporating Ag into their composition. This research highlights the critical importance of evaluating electrode longevity, especially in remote biomedical applications like smart wearables, where electrode performance over time is crucial for reliable and sustained monitoring and stimulation.

## 1. Introduction

For a society in constant development, ensuring the health and well-being of all is a major concern, especially in increasingly aging populations. Moreover, sedentary lifestyles are spreading worldwide, not only because of the lack of available spaces for exercise but also as a result of more occupational sedentary behaviors, such as office work, and the increasing popularity of electronic/video devices (e.g., TVs, computers, mobile phones, etc.). Consequently, the increasing prevalence of lifestyle-related diseases requires the constant monitoring of some simple everyday life activities, such as body movement and walking. In this sense, remote devices have become extremely relevant [1,2]. Biosignal acquisition, particularly through electromyography (EMG), is pivotal for the research, diagnosis, therapy, and the development of new person-centered approaches. EMG electrodes allow the recording of the electrical activity of muscles and nerve cells, enabling the assessment of skeletal muscle health. It plays a crucial role in detecting and treating neuromuscular disorders while advancing state-of-the-art improved patient care [3]. The EMG response is also fundamental for some basic treatments based on functional electrical stimulation (FES), since it provides a qualitative measurement of the adequacy of the parameters of each treatment, monitoring the muscular response and the fatigue. FES is commonly used to treat problems related to the sensory–motor system (e.g., muscle atrophy, joint contractures, cervical injuries, or even be a potential treatment for Parkinson’s disease) [4,5]. During FES, currents with different pulses and frequencies go through electrodes to excite a target muscle or muscle group; the parameters are set for each treatment and can offer a partial restoration of certain body functions [5,6,7,8].

Traditionally, “wet” EMG electrodes, composed of silver/silver chloride (Ag/AgCl), are used as biopotential electrodes by healthcare centers to perform routine exams such as electrocardiograms (ECG), electromyography and electroencephalograms (EEG) [9,10,11]. They are considered the gold standard due to their biocompatibility and stability, which is connected to their low-frequency noise and drift [12,13], offering high-quality signal recordings at low cost [14,15]. However, this type of electrode needs a conductive hydrogel/electrolyte at the interface between the sensor and the skin. This is, most likely, their major downside, since the use of a gel limits the possibility of long-term monitoring and strongly contributes to the increase in hospital residues/wastes with great environmental impact [9,12]. Additionally, changes in the humidity of the skin and perspiration alter the gel characteristics and the electrode/skin impedance, degrading the signal quality and introducing motion artifacts. On top of that, the use of the gel often causes discomfort, irritation, redness, and allergies, especially in people with sensitive skin, such as babies and the elderly [9,16,17]. 

To overcome the drawbacks of wet electrodes, dry electrodes have been progressively investigated in recent years since they can be applied in a user-friendly way due to the lack of preparation for skin contact, and they can be worn for long periods. They are designed with highly conductive and biocompatible materials, establishing a reliable mechanical connection with the skin for signal transduction without the need for an electrolyte [12,14,16]. They are meant to be able to be used for continuous monitoring, making them suited to be integrated into wearables in new e-health solutions [18,19]. On the downside, dry electrodes have a higher impedance at the electrode–skin interface, reducing the amplitude signals, and are more susceptible to motion artifacts compared with wet electrodes [9,16,17,20,21]. 

Furthermore, it has been reported that sweat accumulation in the gap between the skin and electrode significantly reduces the impedance after certain periods. Keeping in mind that sweat is a very corrosive solution, and taking account the combination of the long exposition times to sweat and the combination of skin motion and electrical stimulation currents (in FES cases), it is important to understand how these factors will affect the electrode’s lifespan, since they can compromise the electrode’s contact surface with skin, resulting in signal degradation and loss of confidence in the signal monitored [9,16,22,23]. 

The major problem concerning the use of dry electrodes is related to external factors such as temperature, sweat, electrical currents, and moisture solutions. These factors can, directly or indirectly, greatly affect the biopotential recording precision and the lifespan of the electrodes. Thus, it is crucial to assess their performance under extreme conditions to guarantee their high conductivity and the stability of signal recordings over long monitoring periods [24,25]. 

Some studies have been carried out to determine the lifetime of electrodes exposed to corrosive environments, such as sweat. Kim et al. [23] studied the lifetime of a microneedle array electrode made of polydimethylsiloxane (PDMS) to measure EMG signals; their results showed an 8 h robustness to perspiration, maintaining its high-quality measurement ability. Abdoli-Eramaki and co-authors [22] studied the degradation of Ag/AgCl electrodes for EMG monitoring, concluding that sweat dampened the signal of the electrode; for every sweat layer with 0.02 mm thickness formed, the sample showed a deterioration of 2 to 3%. In this study, dry electrodes tested for long-term biopotential monitoring in prior works were studied in terms of their longevity [10,26,27,28]. The dry electrodes were based on PTFE (polytetrafluoroethylene) substrates functionalized with titanium (Ti) thin films doped with different amounts of silver (Ag). The thin films were prepared by DC magnetron sputtering, using a different number of Ag pellets glued over the erosion zone of the Ti target, enabling the production of four distinct sample sets with different contents of Ag, including a pristine Ti thin film. Ti and Ag were chosen due to their biocompatibility, high conductivity, good mechanical properties, and high corrosion resistance [10,26,29,30]. The electrodes were designed to be integrated into wearables in EMG monitoring and FES stimulation for long periods, aiming at elderly rehabilitation. The lifespan of the electrodes is crucial for the application; therefore, the chemical and physical degradation due to sweat on the electrodes was evaluated. The surface of the electrodes was characterized before and immediately after being in contact with artificial sweat for different periods. In the same way, voltammetry assays were conducted to evaluate the eventual release of Ti or Ag from the film to the sweat solution. It is very well known that bulk Ti exhibits outstanding corrosive resistance. However, the corrosion behavior of Ti-based thin films prepared under non-equilibrium conditions must be assessed to consider the effects of sweat on the electrode’s surface [31,32]. 

## 2. Materials and Methods

### 2.1. Preparation of Ti-Ag Dry Electrodes

For the preparation of the EMG (177 mm^2^ surface area) and FES (314 mm^2^ surface area) dry electrodes, flexible PTFE substrates were functionalized with Ti-Ag thin films. The electrodes were prepared by DC magnetron sputtering at room temperature, using a laboratory-sized deposition system. 

Immediately before the depositions, the PTFE substrates (thickness 0.10 mm, REF. CF905-2. Sove^®^) were activated by low-temperature plasma treatments to improve the adhesion between the polymer surface and the thin film deposited. The substrates were thoroughly cleaned with isopropyl alcohol and then introduced into a plasma cleaner (Plasma System Zepto, Diener electronic GmbH & Co. KG, Ebhausen, Germany) to be treated in an argon (Ar) atmosphere powered at 50 W for 1200 s [26,33].

For the depositions, a Ti target (99.96% purity) with a rectangular geometry (200 × 100 × 6 mm^3^ dimensions) was used. The target was modified with a growing number of Ag metallic pellets (16 mm^2^ area and 0.5 mm thickness) glued onto the erosion zone of the target with conductive Ag paint. 

The depositions occurred at room temperature for very low base pressures (<2.0 × 10^−4^ Pa). During the sputtering, a current density of 75 A/m^2^ was applied to the Ti-Ag-composed target (cathode), whilst a 25 sccm Ar flow was kept constant (work pressure 3.0 × 10^−1^ Pa) inside the chamber. The polymer substrates were fixed into a substrate holder (anode) in the center of the reactor, distanced 70 mm from the cathode. To guarantee homogeneous depositions, the substrate holder was kept in constant rotation with a speed of 5 rpm. The deposition time was fixed at 40 min, and the maximum temperature reached did not exceed 60 °C, ensuring that there was no thermal degradation of the substrates [34].

### 2.2. Ti-Ag Thin Film Characterization

The chemical composition of as-deposited Ti-Ag thin films was analyzed through Rutherford Backscattering Spectrometry (RBS) using a 2 MeV 4He+ ions beam at nearly normal incidence. Three strategically positioned detectors, including one standard detector at 140° and two PIN-diode detectors symmetrically placed at 165°, recorded the backscattered ions. The recorded data were analyzed with NDF algorithms [35,36], allowing the precise determination of the Ti and Ag contents in the films. The crystalline structure and phase distribution of the Ti-Ag thin films were evaluated through X-ray diffraction (XRD) using a D8 Discover diffractometer (Bruker Corp., Billerica, MA, USA). For the analysis, Cu-Kα radiation with a wavelength of 1.5406 Å was used, and the step size was maintained at 0.04° over a 2θ range of 30–80°.

To assess the surface morphology and film growth, a scanning electron microscopy (SEM) analysis was carried out using a NanoSEM-FEI Nova 200 electron microscope from FEI Corporation in Hillsboro, OR, USA, equipped with a field emission gun (FEG). The cross-section micrographs obtained from the SEM analysis were utilized to determine the thickness of the Ti-Me thin films. 

The Ti-Ag thin films were deposited onto monocrystalline silicon substrates (100 P-type/B) with (100) orientation for the RBS, XRD, and SEM analyses.

### 2.3. Ti-Ag Electrode Lifespan

The long-term stability of the different types of Ti-Ag electrodes was assessed under extreme conditions by immersing them in a 20 cm^3^ artificial sweat solution (standard ISO-3160-2). The immersion was carried out in an incubator (LabCompanion IST-4075R) at a temperature of 37 ± 2 °C, maintaining a constant rotational speed of 30 rpm for different periods (1 h, 4 h, 24 h, 168 h, and 240 h). These conditions were chosen to replicate the operating environment of the electrodes. Multiple replicates were used for each immersion period to ensure the accuracy of the data. 

To better evaluate the degradation processes that occurred, the surface of the electrodes was evaluated by surface characterization techniques immediately before and after immersion. 

The optical changes on the surface of the electrodes were examined using an optical microscope (Nikon, Optiphot 100) connected to a CCD camera (Sony, CCD-Iris) with a magnification of 10× and using the Leica Application Suite System software DM2500. Four images were taken from different regions of each electrode. To assess the percentage of defects present on the surface, a MATLAB algorithm software (version 2023a, The MathWorks, Inc., Natick, MA, USA) was utilized.

The chemical changes occurring on the surface of the electrodes were assessed by Fourier transform–infrared spectroscopy (FTIR). The measurements were performed using a Jasco FT/IR 4100 system operating in the attenuated total reflectance (ATR) mode, covering a wavelength range of 4000–600 cm^−1^ with a resolution of 8 cm^−1^ and 64 scans/min. 

Also, the electrical behavior of the electrodes was evaluated by measuring their electrical resistivity at room temperature using the four-point probe method. The measurements were conducted employing the Ossila Four-Point Probe rig (Ossila Ltd., Sheffield, UK). The probe head had four spring-loaded contacts with a diameter of 0.48 mm, spaced 1.27 mm apart, that applied a normal spring pressure of 60 g onto the sample. The electrical resistivity was measured in three different spots on the samples and repeated on two distinct electrodes with the same composition, taking into account the thickness of the Ti-Ag films.

To determine the amount of Ag released from the film into the sweat solution, after the electrode’s immersion, anodic stripping voltametric experiments were performed at room temperature using a potentiostat (Autolab PGSTAT30, Ecochemie) controlled by GPES 4.9 software. A conventional one-compartment cell (v = 10 mL) with a glassy carbon electrode (GCE) as the working electrode (3 mm diameter disk electrode, CHI104, CH Instruments, Inc), a platinum wire as a secondary electrode, and a Ag/AgCl electrode (3 M KCl; CH Instruments, Inc., Cambria, UK) was used as a reference electrode. Before the experiments, the GCE was polished with a polishing cloth (Buehler) with alumina suspension (MicroPolish; Buehler; 0.05 μm). The electrode was thoroughly rinsed with ultrapure water and dried with absorbent paper before recording each voltammogram. 

Anodic stripping voltammograms using the square-wave voltammetry technique were obtained with a deposition time of 120 s, deposition potential of −0.9 V, equilibrium time of 5 s, and stirring at 300 rpm. For square-wave voltammetry, the following experimental parameters were used: frequency of 80 Hz, pulse amplitude of 100 mV and potential step of 2 mV.

## 3. Results

### 3.1. Composition and Microstructural Analysis

Four types of electrodes with distinct compositions were prepared. One was of pure titanium (Ti) while the others, labelled as “Ti-Ag 1”, “Ti-Ag 2”, and “Ti-Ag 3”, contained varying amounts of silver (Ag) at different Ag/Ti ratios. The Ti electrodes were used as a reference and for comparison purposes. The chemical composition of the Ti-Ag thin films was determined by RBS and presented in Table 1. The error associated with the determination of both elements—Ti and Ag—was about 0.5 at.%.

The RBS profiles revealed that the Ti-Ag thin films exhibited uniform in-depth composition profiles. As described in previous works [26,27,28], an increase in the exposed Ag area on the erosion zone of the target leads to higher Ag/Ti ratios in the film. Beyond the influence of all discharge parameters, for the same Ar^+^ energy values, the sputtering yield of Ag was found to be higher than that of Ti [37]. Consequently, an increase in the amount of Ag pellets led to higher Ag contents in the films.

**Figure 1:**
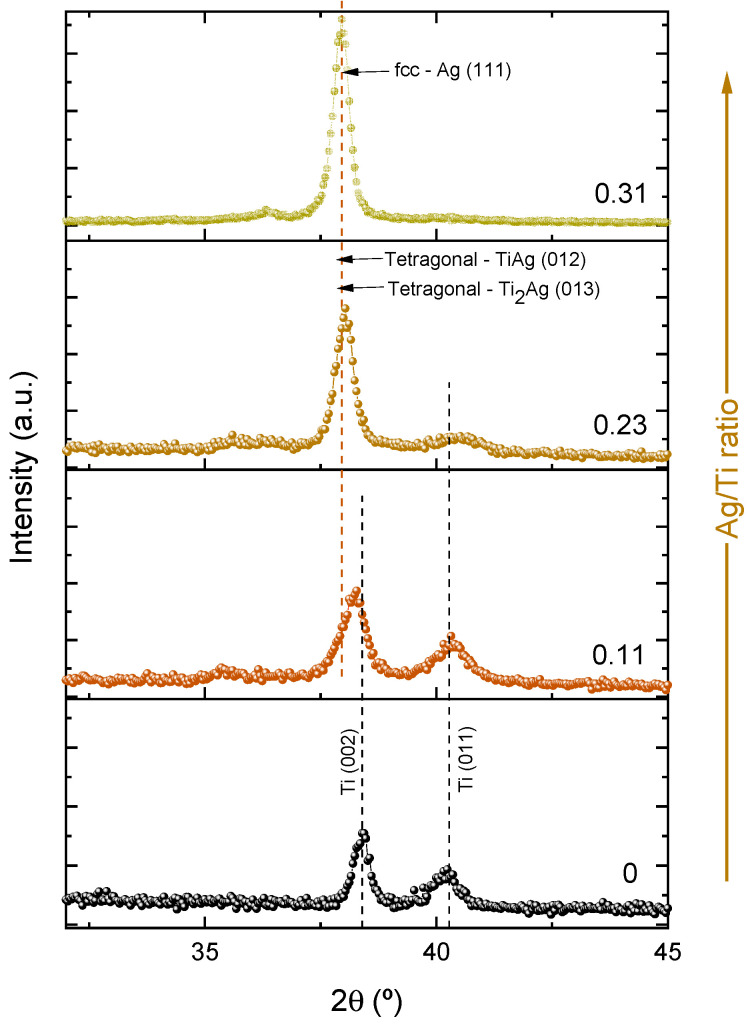
Diffractograms of the thin films studied.

The distinct chemical composition of the Ti-Ag thin films resulted in significant differences in the structural features, as depicted in the X-ray diffractograms presented in Figure 1. For the pure-Ti thin film, it is possible to observe two different Bragg peaks, located at 2θ ≈ 40.59° and 2θ ≈ 38.92°, corresponding, to the (011) and (002) orientations (ICSD card #181718), respectively. These peaks can easily be indexed to the α phase of Titanium’s high close-packed hexagonal structures (hcp). Typically, the (002) orientation is the preferred growth orientation [30,38,39,40].

The thin film with the lowest Ag content (Ag/Ti ratio = 0.11) exhibited a structure closely resembling that of the pure-Ti film. This structural characteristic can be easily understood since Ti is the main component in the film. However, a closer look at Figure 1 shows a slight shift of the diffraction peak located at 2θ ≈ 38.92° to lower values, indicating higher lattice parameters. This deviation might be related to the interstitial diffusion of Ag into the Ti lattice. The depositions were carried out at low temperatures in a non-thermodynamic equilibrium. During this process, sputtered Ag and Ti atoms randomly reach the substrate’s surface with low energies, leading to a high degree of structural disorder and the formation of metastable phases. In less pure α-Ti structures, interstitial-related diffusion mechanisms tend to occur more rapidly and with lower activation energies, particularly when metallic impurity atoms have smaller atomic radii compared with the unusually open structure of α-Ti [41]. Both Ti and Ag atoms have similar atomic radii (0.144 nm and 0.145 nm, respectively [42]). Consequently, Ag atoms are “dissolved” into the α-Ti matrix. The interstitial diffusion occurs when Ag atoms occupy the interstitial spaces (gaps) between the Ti atoms in the lattice. These gaps are typically larger than the atomic radius of silver. Unlike substitutional diffusion, interstitial diffusion is faster and can occur at lower temperatures because it does not require the disruption of existing bonds within the lattice [43]. The film revealed characteristics of a solid solution, with silver assuming the role of the solute within the titanium matrix (solvent). In this context, it seems that the Ag atoms are uniformly dispersed within the Ti lattice, forming a homogeneous and integrated mixture at the atomic level.

As the Ag content increases in the films (Ag/Ti ratios ≥ 0.23), significant changes in the diffraction patterns become evident. The previously main preferential orientation (002) of the Ti structure shifts from 2θ ≈ 38.92° to 2θ ≈ 38.09°, becoming more intense and sharper. This shift might be attributed to the precipitation of tetragonal Ti-Ag intermetallic phases into the Ti matrix, such as TiAg (ICSD #44872) and/or Ti_2_Ag (ICSD #605931), in agreement with the binary Ti-Ag equilibrium phase diagram and previous works [26,27,44]. However, the thin films were not prepared in thermodynamically favorable conditions. It is possible that the peak at 2θ ≈ 38.09° can also include the diffraction of the (111) pure fcc-Ag crystal phase, diffracting at 2θ ≈ 38.11°, according to the ICSD #181730. Additionally, the diffraction peak with the (011) orientation becomes broader and less evident, indicating a trend toward the amorphization of the Ti structure. Even though the α-Ti phase cannot be detected by X-ray diffraction, its significance should not be underestimated, as Ti remains the predominant component in the films. Nevertheless, it is obvious that the presence of higher amounts of silver affects the crystalline structure of titanium, leading to a loss of the (011) orientation and a more disordered arrangement in the film prepared with the highest Ag content (Ag/Ti ratio of 0.31). The presence of Ti-Ag intermetallic phases, evidenced by the diffractograms, highlights the interstitial diffusion processes that occurred during the deposition [45]. Interstitial diffusion is closely related to the formation of intermetallic compounds. In this case, Ag atoms occupy interstitial sites, establishing new bonds with Ti, and the hcp-Ti crystal structure tends to evolve into the Ti-Ag tetragonal structure (I4/mmm space group).

**Figure 2:**
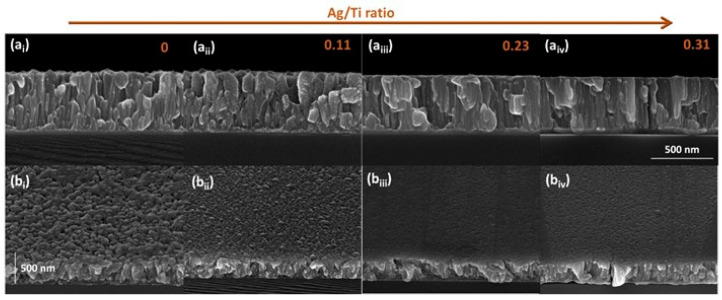
Cross-sectional images (**a_i_**–**a_iv_**) and top-view images (**b_i_**–**b_iv_**) of the Ti-Ag thin films produced with different Ag/Ti ratios.

The results of the addition of Ag to the Ti-matrix in the columnar growth and on the topography exhibited by the Ti-Ag films are presented in Figure 2. Based on the cross-section micrographs in Figure 2(a_i_–a_iv_), it is clear that all the films exhibit strong adhesion to the substrate and have very similar thicknesses. Although it was expected that increasing the Ag/Ti ratio would lead to thicker films due to the high Ag sputtering yield, all the Ti-Ag films were consistently prepared with a thickness of approximately 500 nm. A plausible explanation for this observation could be attributed to the increase in the films’ density, which is evidenced by the microstructural growth in Figure 2. As the Ag content increases, the columnar growth of the pure-Ti thin film gives rise to denser microstructures with a higher material content per unit volume, while the overall thickness remains unchanged.

The influence of the Ag addition is also quite noticeable in the topography of the films, as shown in Figure 2(b_i_–b_iv_). As the Ag content increases, the films reveal smoother surfaces, which is in correlation with the dense columns observed in the cross-sectional images. As previously mentioned, pure-Ti thin films typically grow in the α-phase, resulting in a rough surface morphology with three-dimensional hexagonal grain features [28,38,39]. However, the incorporation of Ag leads to smoother surfaces, which are particularly noticeable in the samples prepared with Ag/Ti ratios of 0.23 and 0.31. These findings are consistent with prior research [26], validating the consistency of the obtained results.

### 3.2. Ti-Ag Electrode Lifespan

Assessing the lifespan of dry electrodes is crucial for ensuring reliable and accurate applications of electromyography (EMG) and functional electrical stimulation (FES). This is especially significant for surface electrodes used in challenging environments like human transpiration. The Ti-Ag electrodes were evaluated for their long-term viability, stability, and signal quality, considering the various microstructures prepared using different Ag/Ti ratios. Two approaches were employed for this assessment: one examines the electrode’s surface that will be in contact with the skin, while the other analyzes the material release and wear from exposure to sweat solution.

#### 3.2.1. Optical Evaluation

**Figure 3:**
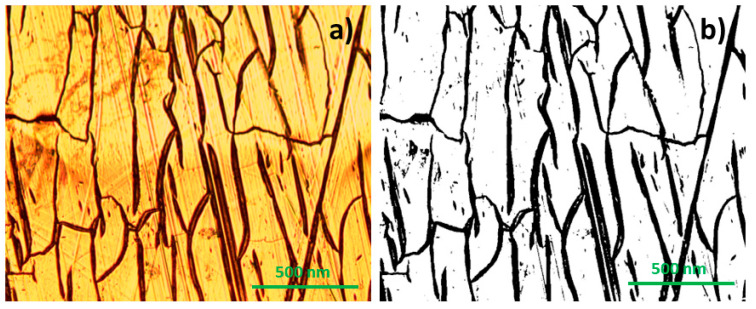
Optical defects observed on the surface of an electrode with an Ag/Ti ratio of 0.31. (**a**) Image recorded by the optical microscope. (**b**) Image created by the MATLAB algorithm, marking the defects (black lines).

The surface of the electrodes was analyzed using optical microscopy since no noticeable changes were detected through a visual inspection. The number of defects detected on each electrode was quantified using a MATLAB algorithm before and immediately after their immersion into artificial sweat. To quantify the surface defects, the algorithm generated an image where the black regions are the imperfections/inhomogeneities detected at the surface. Subsequently, the software calculated the proportion of the dark area relative to the total surface analyzed, representing this ratio as a percentage. Figure 3 shows a visual representation of the defects observed on the surface of an electrode with an Ag/Ti ratio = 0.31, after immersion in sweat solution.

**Figure 4:**
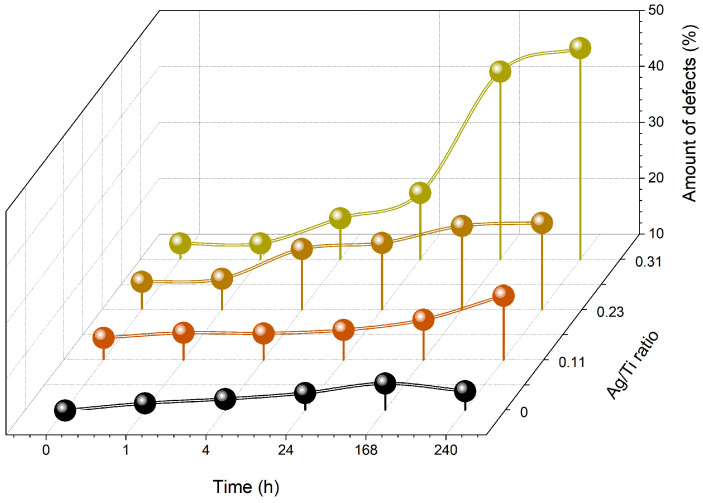
The percentage of surface defects on the electrodes after varying immersion durations in an artificial sweat solution.

Figure 4 depicts the percentage of defects detected on the surface of each type of electrode prepared, after immersion in artificial sweat for different times. The results show that, regardless of the composition of the Ti-Ag thin films, after 1 h of immersion, no significant changes were detected on the electrode´s surface. It is also possible to observe that for the electrodes prepared with Ag, the number of defects on the electrode´s surface increases with the immersion time. 

After 24 h immersed in artificial sweat, the electrodes with higher Ag content reveal a significant increase in the percentage of surface defects, rising from 22 to 44%. This value was significantly higher compared with the electrodes prepared with intermediate Ag content (Ag/Ti = 0.22), which did not exceed 25% of defects on the surface. The results suggest the degradation of the films richer in Ag, which can severely compromise the electrodes’ function. According to the literature, Ag is easily corroded in environments containing chloride ions, which is the case in this study, since artificial sweat solution contains sodium chloride [46]. This is due to pitting, a form of localized corrosion that causes metal destruction by aggressive ions like Cl^−^ [47]. In samples with low Ag content, the first signs of degradation occur after 168 h. From chemical and microstructural analyses, it was found that the electrodes prepared with Ag/Ti ratios of 0.11 structurally behave like the reference electrode of pure Ti. In fact, for the Ti reference sample, only a slight increase in the percentage of defects could be observed after 168 h immersion. Additionally, after 10 days of immersion (240 h), the percentage of surface defects on the Ti electrodes decreases, which may suppose a surface stabilization. The literature reports that Ti oxidation leads to a stable form of TiO_2_, formed by the passivation processes. This phenomenon is responsible for the excellent Ti corrosion resistance [48].

#### 3.2.2. Chemical Analysis

FTIR was employed to evaluate the chemical modifications promoted on the different Ti-Ag electrodes after immersion in artificial sweat for different periods. The FTIR analysis provides a measure of the bonding mechanisms occurring on the surface of the electrodes, adding extra information about the degradation processes [49]. The biopotential electrodes are a result of a polymeric substrate coated with a Ti-Ag thin film. Thus, if the spectrum of the polymeric surface becomes detectable, it indicates the existence of corrosion or degradation phenomena on the film. The film is no longer impermeable to the IR beam (depth 0.2 to 5 µm) [50,51], implying that the columnar-dense morphology features have been altered or destroyed. 

**Figure 5:**
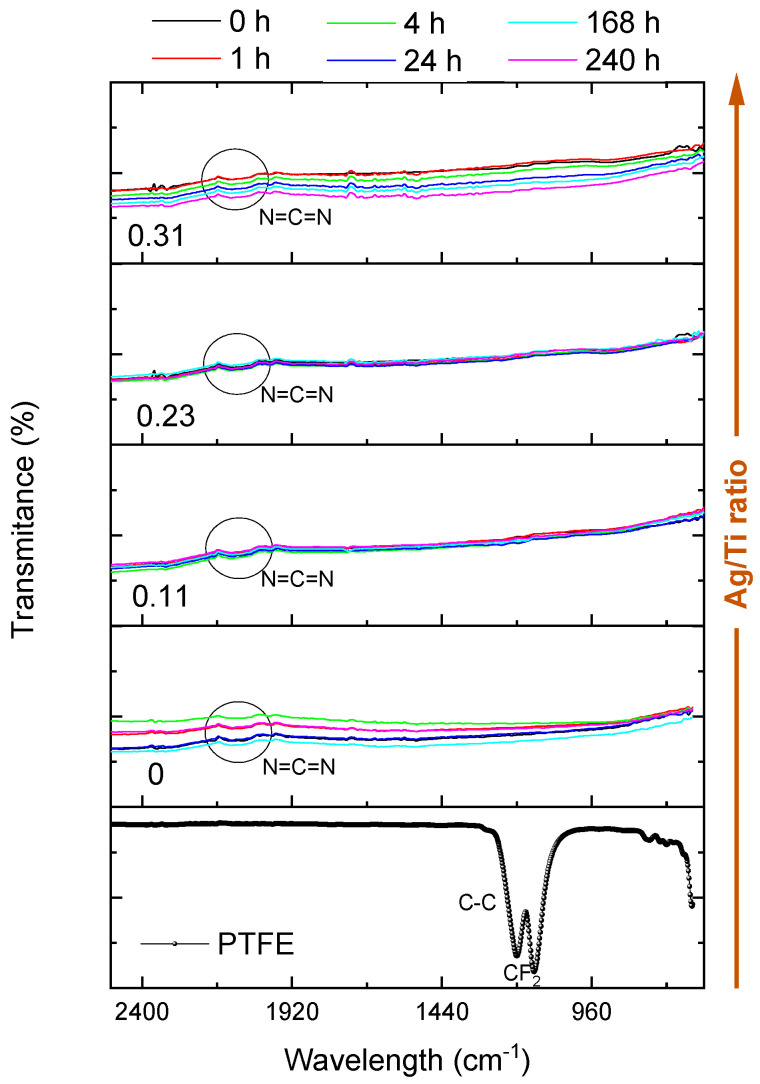
FTIR spectra of the electrodes before and after immersion in artificial sweat solution and of PTFE.

Figure 5 presents the results of the FTIR analysis conducted on Ti-Ag electrodes in the ATR mode. 

One representative spectrum of each sample’s composition and immersion time was selected. The transmittance spectrum of the PTFE substrate was added for comparison purposes. 

The PTFE substrate exhibits distinct bands which correspond to CF_2_ vibrations at approximately 1150 cm^−1^ and 1210 cm^−1^ (symmetric and antisymmetric bands, respectively), with the last one overlapping the C-C band at around 1240 cm^−1^. Additionally, CF_2_ vibrations at around 640 cm^−1^ can also be observed [52]. The analysis reveals that, regardless of the type of electrode, all the spectra are quite similar, and no degradation effects (chemical changes) were noticed. Nevertheless, all the electrodes immersed in sweat solution revealed minor traces of a broad absorption band at around 2100 cm^−1^, which may be linked to the presence of a carbodiimide bond (N=C=N). Song et al. [29] discovered that urea, which constitutes 0.1% (mass percentage) of the sweat solution, was chemically adsorbed onto a Ni-P coating, which might also be happening in these electrodes that were prepared with Ti-Ag thin films. Thus, the minor traces of this band at 2100 cm^−1^ might be due to the formation of carbodiimides resulting from urea dehydration. The Ti-Ag electrodes’ FTIR spectra did not reveal evident signs of degradation, since the CF_2_ bonds of the PTFE substrate were not detected. Nevertheless, the absence of degradation cannot be claimed. Considering that the Ti-Ag thin films’ thickness was around 500 nm, below the laser’s penetration depth, the results revealed that, despite the degradation discussed earlier, the films retained their IR impermeability.

#### 3.2.3. Electrical Behavior

**Figure 6:**
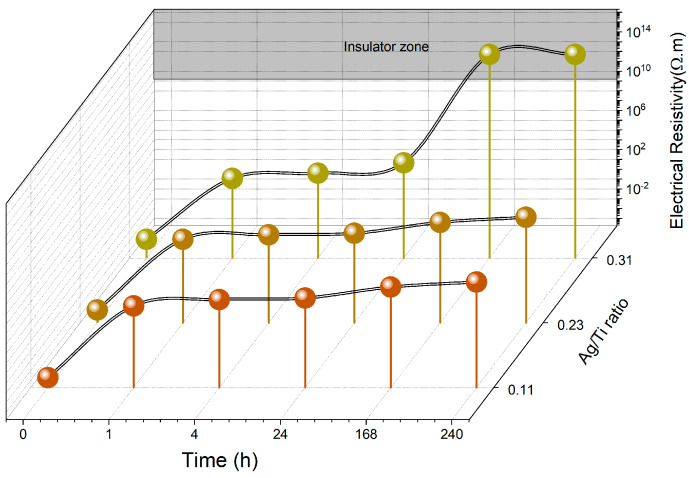
Variation of Ti-Ag electrodes’ electrical resistivity after immersion in artificial sweat for different times.

The electrical properties of the electrodes prepared are crucial for ensuring both biopotential monitoring and electrical stimulation. Therefore, the primary objective of this study was to comprehend the degradation effects on the electrodes’ electrical conductivity. Figure 6 shows the changes in electrical resistivity for each type of electrode prepared before (0 h) and after degradation (immersion in artificial sweat at 37 °C with constant stirring). The graph does not include the Ti electrodes, since after being immersed in sweat, the readings surpassed the electrical resistivity of 10^12^ Ωm, becoming insulators. 

This observation becomes particularly significant if we consider the possible formation of a thin but highly resistive TiO_2_ layer [53,54] on the surface of the Ti electrodes due to the passivation phenomenon. The resistivity of TiO_2_ thin films in different crystallinity degrees was measured by Rao et al. [55], reaching values of around 4.46 × 10^13^ Ωm and 1.98 × 10^14^ Ωm, which support the formation of a TiO_2_ layer onto the immersed Ti electrodes.

The electrical resistivity data for all the Ti-Ag electrodes show a clear trend. Upon immersion into artificial sweat, a substantial increase in resistivity is observed. This notable rise in electrical resistivity becomes evident within just 1 hour of immersion, particularly for electrodes with lower and intermediate Ag contents (Ag/Ti ratios of 0.11 and 0.23, respectively). Remarkably, even with immersion periods lasting up to 24 h, the variation in electrical resistivity remains below 10^3^ Ωm, ensuring that these electrodes maintained their operational capability [26]. Only after an extended immersion period of 7 days did the electrodes display significant degradation effects, raising the electrical resistivity to values of around 10^5^ Ωm.

For the Ti-richer electrodes (Ag/Ti = 0.11), the electrical behavior appears to be correlated with the formation of a TiO_2_ passivation layer, which becomes sufficiently thick enough after 7 days to increase the electrode’s impedance. On the other hand, for the electrodes prepared with an Ag/Ti ratio of 0.23, the observed electrical stability could be attributed to the formation of stable intermetallic phases, as discussed in Section 3.1.

Interestingly, the increasing number of defects observed with immersion time, as shown in Figure 4, is reflected in an increase in electrical resistivity. The electrical conduction in metallic systems, especially in thin films, is highly sensitive to the material’s composition and microstructural features. Factors such as scattering processes on the surface and contributions from defects and impurities within the crystalline structure significantly influence charge carrier conduction, as observed in [56,57].

The electrodes with the highest Ag/Ti ratio exhibited a quite different trend. Differently from their counterparts, after 1 h of degradation, the electrical resistivity of these electrodes had risen to around 15 Ωm, showing very interesting electrical conductive properties. In this study, these electrodes were prepared with the highest amount of Ag, which is very well known for its exceptional electrical conductivity. The Ag-richer phases evidenced by the X-ray diffraction of these electrodes (Figure 1) may be responsible for their tailored electrical behavior [30]. However, over longer immersion periods, the degradation’s impact on the electrodes becomes significantly more apparent, leading to a substantial rise in their electrical resistivity. After 24 h, these electrodes reached their operational limit, and within 168 h, they became insulators. In fact, and in agreement with the surface defects (Figure 4), after 7 days of being immersed in sweat, these electrodes revealed the presence of 50% of defects on the analyzed surface area. The previously prominent role of Ag in facilitating high conductivity is no longer evident, suggesting that Ag may be released from the thin film into the solution during degradation.

#### 3.2.4. Voltammetry Analysis

**Figure 7:**
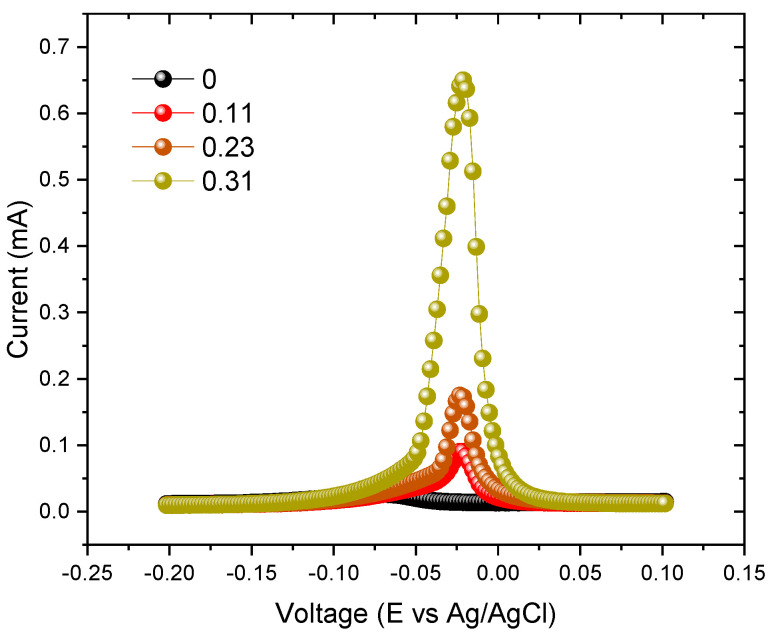
Anodic stripping voltammograms of the different electrodes immersed in the sweat solution for 240 h, using the square-wave voltammetry technique with a deposition time of 120 s, a potential of −0.9 V, an equilibrium time of 5 s, and stirring at 300 rpm with a GCE electrode.

**Figure 8:**
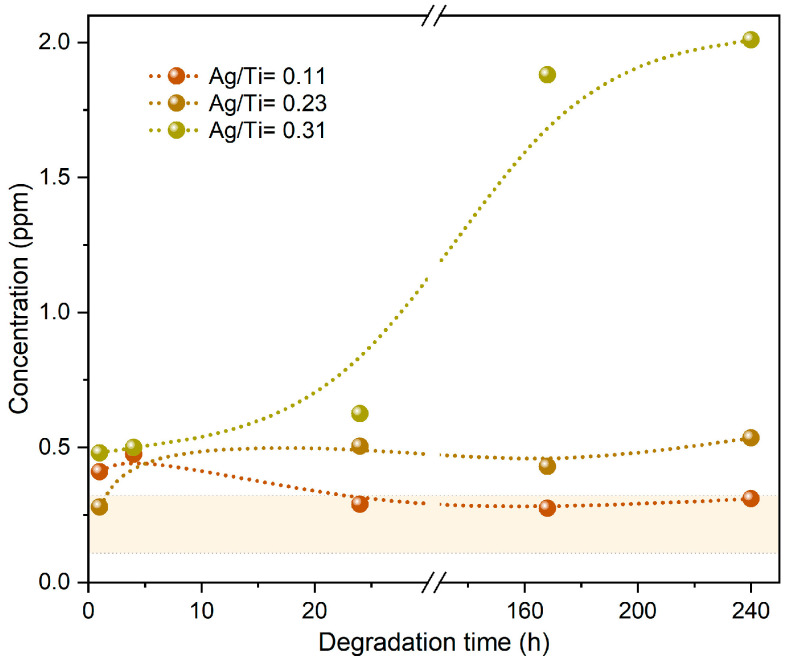
Concentration of Ag released into the artificial sweat solution after immersion. The values represent the average of the replicates over time.

The voltammetry analysis was conducted on all the sweat solutions used for immersing the various types of electrodes over time. This analysis enabled the quantification of the Ag content that was released from the thin film into the sweat solution. In each experiment, whether involving standard solutions or solutions in contact with the electrodes, three voltammograms were recorded. The voltammograms exhibited very good precision, consistently maintaining a relative standard deviation below 5%. Figure 7 depicts the voltammetric response acquired from the sweat solution after immersing the electrodes for 240 h. The results of this analysis are visually represented in Figure 8, providing valuable insights into the extent of Ag release and its correlation with the immersion period.

In the graph, the shaded area represents the region bounded by a limit of detection (LOD) and a limit of quantification (LOQ). The LOD, defined at one-third of the LOQ is 0.033 ppm, while the LOQ is 0.100 ppm for the determination of Ag. While the LOD represents the minimum concentration that the method can detect, the LOQ represents the minimum concentration that the method can quantify rigorously [58]. So, the results indicate that for lower immersion periods (1 h, 4 h, and 24 h), the presence of Ag can be detected, but its exact quantification might not be precise. 

Furthermore, for the electrodes with the lowest Ag content (Ag/Ti = 0.11), it was challenging to precisely determine the amount of Ag in the sweat solution, since it is below the LOQ line, even after a 10-day period. According to the previous results, the Ag contained within the films used to coat these electrodes is entrapped in the hcp α-Ti structure, hindering its release into the sweat solution. For these electrodes, the degradation effects are mostly related to the Ti behavior and are thus less pronounced. 

Also, the electrodes with an Ag/Ti ratio of 0.23 exhibited minimal release of Ag, which could only be quantified after 10 days of immersion. Once again, this was an expected result, according to the previous analysis, and can be attributed to the stability of the Ti-Ag intermetallic phases.

The results provide clear evidence that the poor electrical behavior observed in the electrodes with Ag/Ti = 0.31 after 7 days of degradation is directly related to the release of almost all the Ag content into the solution. As previously mentioned, chloride ions play a significant role in Ag corrosion, and in the films with Ag richer phases, this phenomenon becomes more pronounced, reducing the lifetime of these electrodes.

## 4. Conclusions

This study provides valuable insights into the degradation behavior and lifetime of Ti-Ag dry electrodes, deposited by DC magnetron sputtering onto PTFE polymers. The findings contribute to the development of durable and reliable electrodes for long-term EMG monitoring and FES applications, particularly in the context of elderly rehabilitation.

The electrodes were prepared with different Ag/Ti ratios, corresponding to the increased Ag exposed area on the Ti target, as confirmed through the chemical composition analysis. Structural results unveiled a notable evolution from hcp α-Ti structures to the formation of Ti-Ag intermetallic phases and, finally, to fcc-Ag richer phases upon increasing the Ag content in the Ti matrix. The SEM analysis demonstrated that higher Ag contents in the electrodes resulted in the generation of more compact microstructures and smoother surfaces.

The microstructural features developed by the electrodes were highly reflected in their degradation responses. 

The electrodes prepared with the highest Ag/Ti ratio revealed a considerable decline in their electrical performance with prolonged exposure to the artificial sweat. The large number of defects observed on the surface was closely related to significant Ag release due to the corrosion driven by the sweat solution. These electrodes cannot be used for periods longer than 1 day. 

By contrast, the electrodes prepared with lower and intermediate Ag contents (Ag/Ti = 0.11 and Ag/Ti = 0.23) showed a slight increase in electrical resistivity after being immersed in sweat, being still stable and constant for up to 24 h. However, after 168 h, a decline in electrical behavior is noticed, indicating that all the prepared electrodes no longer functioned properly beyond that time. Nonetheless, the electrodes with Ag/Ti ratios of 0.11 and 0.23 demonstrated the highest resistance to sweat environments, making them ideal candidates for extended usage periods in practical applications.

## Figures and Tables

**Table 1:** Composition of the Ti-Ag electrodes, obtained by RBS measurements.

Sample	Nº of Ag Pellets	Pellet Area (cm^2^)	Ti (at.%)	Ag (at.%)	Ag/Ti
Ti	0	0	100	0	-
Ti-Ag1	14	2.2	89.9	10.1	0.11
Ti-Ag2	28	4.5	81.6	18.4	0.23
Ti-Ag3	41	6.6	76.3	23.7	0.31

## Data Availability

Data is unavailable due to privacy restrictions.
